# Pirfenidone, an Anti-Fibrotic Drug, Suppresses the Growth of Human Prostate Cancer Cells by Inducing G_1_ Cell Cycle Arrest

**DOI:** 10.3390/jcm8010044

**Published:** 2019-01-04

**Authors:** Kenichiro Ishii, Takeshi Sasaki, Kazuhiro Iguchi, Manabu Kato, Hideki Kanda, Yoshifumi Hirokawa, Kiminobu Arima, Masatoshi Watanabe, Yoshiki Sugimura

**Affiliations:** 1 Department of Nephro-Urologic Surgery and Andrology, Mie University Graduate School of Medicine, Tsu, Mie 514-8507, Japan; t-sasaki@clin.medic.mie-u.ac.jp (T.S.); katouuro@clin.medic.mie-u.ac.jp (M.K.); kanda331@clin.medic.mie-u.ac.jp (H.K.); kiminobu@clin.medic.mie-u.ac.jp (K.A.); sugimura@clin.medic.mie-u.ac.jp (Y.S.); 2 Department of Oncologic Pathology, Mie University Graduate School of Medicine, Tsu, Mie 514-8507, Japan; ultray2k@doc.medic.mie-u.ac.jp (Y.H.); mawata@doc.medic.mie-u.ac.jp (M.W.); 3 Laboratory of Community Pharmacy, Gifu Pharmaceutical University, Gifu, Gifu 501-1196, Japan; iguchi@gifu-pu.ac.jp

**Keywords:** prostate cancer, androgen sensitivity, pirfenidone, TGFβ1, G_1_ cell cycle arrest

## Abstract

Pirfenidone (PFD) is an anti-fibrotic drug used to treat idiopathic pulmonary fibrosis by inducing G_1_ cell cycle arrest in fibroblasts. We hypothesize that PFD can induce G_1_ cell cycle arrest in different types of cells, including cancer cells. To investigate the effects of PFD treatment on the growth of human prostate cancer (PCa) cells, we used an androgen-sensitive human PCa cell line (LNCaP) and its sublines (androgen-low-sensitive E9 and F10 cells and androgen-insensitive AIDL cells), as well as an androgen-insensitive human PCa cell line (PC-3). PFD treatment suppressed the growth of all PCa cells. Transforming growth factor β1 secretion was significantly increased in PFD-treated PCa cells. In both LNCaP and PC-3 cells, PFD treatment increased the population of cells in the G_0_/G_1_ phase, which was accompanied by a decrease in the S/G_2_ cell population. CDK2 protein expression was clearly decreased in PFD-treated LNCaP and PC-3 cells, whereas p21 protein expression was increased in only PFD-treated LNCaP cells. In conclusion, PFD may serve as a novel therapeutic drug that induces G_1_ cell cycle arrest in human PCa cells independently of androgen sensitivity. Thus, in the tumor microenvironment, PFD might target not only fibroblasts, but also heterogeneous PCa cells of varying androgen-sensitivity levels.

## 1. Introduction

The number of males diagnosed with prostate cancer (PCa) is increasing worldwide [1]. Most patients with early-stage PCa can be treated with therapies such as radical prostatectomy or irradiation, whereas androgen deprivation therapy (ADT) is the standard systemic therapy given to patients with advanced PCa. Even though ADT induces temporary remission, the majority of patients (approximately 60%) eventually progress to castration-resistant PCa (CRPC), which is associated with a high mortality rate [2,3].

PCa is characterized by multifocal and heterogeneous progression of the primary tumor. In PCa progression, a decrease or loss of androgen sensitivity in PCa cells is a significant clinical concern. CRPC, a heterogeneous disease, exhibits varying degrees of androgen sensitivity. Once PCa cells lose sensitivity to ADT, effective therapies are limited [4]. In the past few years, however, several new options for the treatment of CRPC have been approved, including CYP17 inhibitors, androgen receptor (AR) antagonists, and taxane [5]. Despite progress in the development of new drugs, there is a strong medical need to optimize the sequence and combination of approved drugs.

Drug repositioning or repurposing is the process of finding new uses for existing drugs [6], provided that additional clinical trials are relatively easy to perform, and the drug safety profiles have been established. In PCa, there have been a number of drug repositioning studies of non-cancer drugs, including the antidiabetic drug troglitazone, which is a ligand for peroxisome proliferator-activated receptor gamma [7]; the antihypertensive drug candesartan, which is an angiotensin II receptor blocker [8]; naftopidil, which is a selective α_1_-adrenoceptor antagonist used to treat benign prostatic hyperplasia [9]; and the antiallergy drug, tranilast [10]. A drug repositioning approach helps identify new pharmaceutical processes to transform existing drugs into useful sources of new anticancer drugs [11]. 

Pirfenidone (PFD) is an established anti-fibrotic and anti-inflammatory drug used to treat idiopathic pulmonary fibrosis, an interstitial lung disease characterized by accumulation of fibroblasts/myofibroblasts, excessive extracellular matrix production, and altered transforming growth factor β (TGFβ)/bone morphogenetic protein signaling [12,13]. A number of studies have reported that PFD treatment suppresses the growth of and induces G_1_ cell cycle arrest in stromal cells, rat hepatic stellate cells [14], and human Tenon fibroblasts [15,16]. Interestingly, PFD treatment has also been reported to suppress the growth of epithelial cells/cancer cells, including human lens epithelial cells [17] and human hepatocellular carcinoma cells [18]. Epidemiologically, Miura et al. reported a reduced incidence of lung cancer in patients with idiopathic pulmonary fibrosis treated with PFD [19]; however, the mechanism of PFD-induced cancer cell suppression is not well characterized. 

Many studies on CRPC have used androgen-insensitive PCa cell lines, such as PC-3 and DU145 cells, which do not express AR [20]. These cell lines were derived from highly anaplastic tumors from different metastatic sites in the bone and brain [21,22]. The PC-3 and DU145 cell lines both differ strongly in aggressiveness compared with the androgen-sensitive, AR-positive LNCaP cell line, which was derived from a lymph node metastasis [23]. Comparisons between androgen-sensitive LNCaP cells and androgen-insensitive PC-3 and DU145 cell lines may not be relevant to the acquisition of androgen insensitivity in clinical PCa, because many clinical androgen-insensitive PCa cases express AR. A more accurate model of clinical cancer requires, at the very least, an androgen-insensitive, AR-positive cancer cell line. To compare the biochemical characteristics of androgen-insensitive and sensitive PCa cells, we generated three sublines from androgen-sensitive LNCaP cells: E9 and F10 (androgen-low-sensitive) and AIDL (androgen-insensitive) cells [24,25,26]. The parental LNCaP cell line and its derivative E9, F10, and AIDL sublines express similar levels of the AR protein, but androgen-dependent secretion of the prostate-specific antigen (PSA) is only detected in LNCaP cells [27]. In this study, we used the LNCaP cell line and its sublines to investigate the effects of PFD treatment on the growth of human PCa cells, focusing on androgen sensitivity.

## 2. Materials and Methods

### 2.1. Materials

PFD was purchased from Tokyo Chemical Industry Co., Ltd. (Tokyo, Japan). Rabbit monoclonal anti-p21 and anti-CDK2 antibodies were purchased from Cell Signaling Technology (Danvers, MA, USA). Rabbit polyclonal anti-phospho-Akt (Ser473) and anti-Akt antibodies were purchased from Cell Signaling Technology. Mouse monoclonal anti-β-actin (clone AC-15) antibody was purchased from Sigma-Aldrich Co. (St. Louis, MO, USA). Rabbit polyclonal anti-PSA antibody was purchased from Dako Cytomation (Copenhagen, Denmark). Rabbit polyclonal anti-AR (N-20) antibody was purchased from Santa Cruz Biotechnology (Santa Cruz, CA, USA).

### 2.2. Cell Culture

The androgen-sensitive, AR-positive human PCa cell line LNCaP, and the androgen-insensitive, AR-negative human PCa cell line PC-3 were obtained from the American Type Culture Collection (Manassas, VA, USA). LNCaP and PC-3 cells were authenticated by the short tandem repeat method and cultured in RPMI 1640 medium (Nacalai Tesque, Kyoto, Japan) supplemented with 10% fetal bovine serum (Sigma-Aldrich Co.). Androgen-low-sensitive E9 and F10 cells were established from androgen-sensitive LNCaP cells using a limiting dilution method under regular culture conditions [24,25]. In contrast, androgen-insensitive AIDL cells were established from LNCaP cells by continuous passaging under hormone-depleted conditions [26]. The androgen sensitivity of the parental LNCaP cells and the E9, F10, and AIDL cells were confirmed by the change in *KLK3* (PSA) mRNA expression after treatment with the synthetic androgen R1881 [20].

### 2.3. Cell Viability Assay

To assess cell viability after the PFD treatments, LNCaP, E9, F10, AIDL, and PC-3 cells were plated in 12-well plates at 5 × 10^4^ to 1 × 10^5^ cells/well. PFD (0.1 and 0.3 mg/mL) or vehicle-only (0.1% dimethyl sulfoxide [DMSO]) was added on day two, and the cells were cultured for an additional three days. The cells were detached by trypsinization and counted using the Countess II Automated Cell Counter (Thermo Fisher Scientific Inc., Waltham, MA, USA). Cell viability was assessed by trypan blue exclusion assay.

### 2.4. Cell Cycle Analysis

LNCaP or PC-3 cells (1.5 × 10^5^ cells) were seeded into 100-mm culture dishes (Sumitomo Bakelite Co., Ltd., Tokyo, Japan). Twenty-four hours after seeding, the cells were treated with 0.1 or 0.3 mg/mL PFD or vehicle (0.1% DMSO) for 24 h. After treatment, the cells were isolated, and the nuclei were stained using the BD Cycletest Plus DNA Reagent Kit (BD Biosciences, San Jose, CA, USA). To determine the cell cycle distribution, the DNA content of the stained cells was analyzed using the BD FACS Canto II flow cytometer (BD Biosciences), as described previously [28].

### 2.5. Apoptosis Assay

LNCaP cells (6 × 10^5^ cells) and PC-3 cells (4 × 10^5^ cells) were seeded in 100 mm culture dishes (Sumitomo Bakelite Co., Ltd.). 24 h after seeding, the cells were treated with 0.1 or 0.3 mg/mL PFD, or vehicle (0.1% DMSO), for 48 h (LNCaP cells) or 72 h (PC-3 cells). After treatment, the cells were trypsinized, collected, and stained with annexin V–fluorescein isothiocyanate and propidium iodide simultaneously using the Annexin V-FITC Apoptosis Detection kit (BD Biosciences). The cell suspensions were analyzed using the BD FACS Canto II flow cytometer (BD Biosciences) to determine the percentage of apoptotic (annexin V–fluorescein isothiocyanate staining) and necrotic (propidium iodide staining) cells, as described previously [28]. A minimum of 20,000 cells were collected for all samples.

### 2.6. ELISA

For quantitative determination of TGFβ1 and PSA proteins, aliquots of conditioned medium from PCa cells were collected and subjected to ELISA using the Quantikine^®^ human TGF-β1 immunoassay kit (R&D Systems, Inc., Minneapolis, MN, USA) and PSA Enzyme Immunoassay Test Kit (Hope Laboratories, Belmont, CA, USA), respectively.

### 2.7. Preparation of Cell Lysates

LNCaP or PC-3 cells (1 × 10^6^) were seeded in 100 mm culture dishes (Sumitomo Bakelite Co., Ltd.). 24 h after seeding, the cells were treated with PFD (0.1 or 0.3 mg/mL) or vehicle (0.1% DMSO) for 48 h. The cells were harvested by scraping, and whole cell lysates were prepared as described previously [27]. Briefly, the cells were washed with ice-cold phosphate-buffered saline and lysed with CelLytic^TM^ (Sigma-Aldrich Co.) containing 1% Nonidet P-40, 10 mM 4-(2-aminoethyl) benzensulfonyl fluoride, 0.8 mM aprotinin, 50 mM bestatin, 15 mM E-64, 20 mM leupeptin, and 10 mM pepstatin. After 60 min on ice, the lysates were centrifuged at 10,000 *g* for 10 min, and the supernatants were collected. The protein concentration was measured using the NanoDrop 2000 instrument (Thermo Fisher Scientific Inc.).

### 2.8. Western Blot Analysis

Extracted proteins were separated by gel electrophoresis and transferred to Immobilon polyvinylidene difluoride membranes (Merck Millipore, Darmstadt, Germany) following our previously reported protocol [27]. The anti-AR, anti-PSA, anti-phospho-Akt (Ser473), anti-Akt, and anti-β-actin antibodies were used at dilutions of 1:2500, 1:5000, 1:1000, 1:1000, and 1:5000, respectively. Specific protein bands were visualized using the SuperSignal^TM^ West Pico Chemiluminescent Substrate (Thermo Fisher Scientific Inc.) with the LAS-4000 Mini (Fuji Photo Film, Tokyo, Japan). 

### 2.9. Statistical Analysis

Results are expressed as means ± standard deviation. Differences between two groups were determined using Student’s *t*-test. Values of *p* < 0.05 were considered statistically significant.

## 3. Results

### 3.1. Effects of Pirfenidone Treatment on the Growth of Prostate Cancer Cells (LNCaP, LNCaP Sublines, and PC-3)

**Figure 1:**
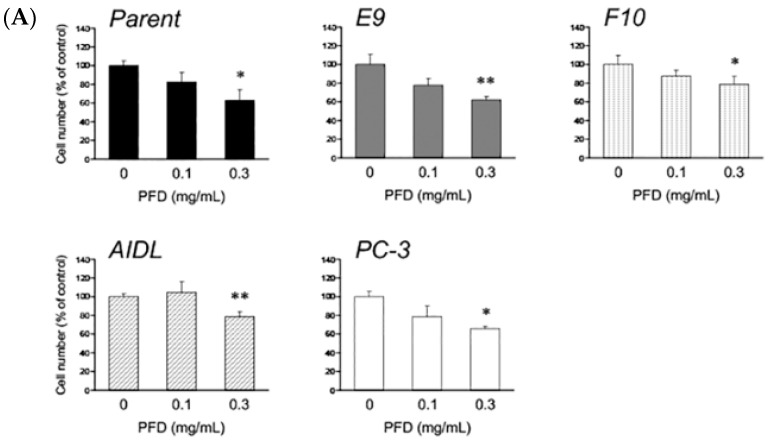

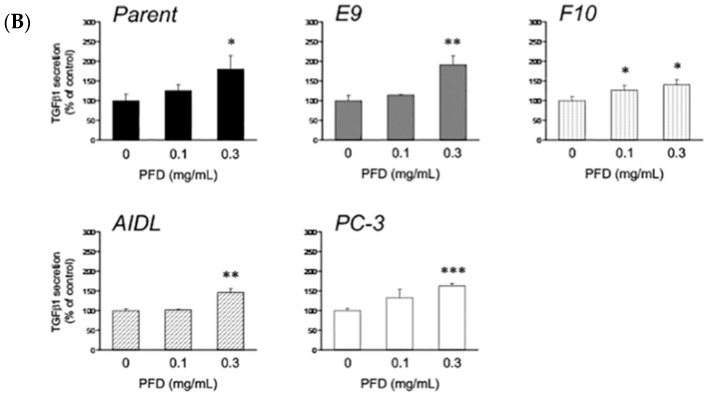
Effects of pirfenidone (PFD) treatment on the growth and secretion of transforming growth factor β1 (TGFβ1) of human prostate cancer (PCa) cells. Human PCa cells (parental LNCaP cell line and the LNCaP sublines E9, F10, and AIDL; and the PC-3 cell line) were plated in 12-well plates and treated with PFD for three days. Effects of PFD treatment on the (**A**) growth and (**B**) TGFβ1 secretion of human PCa cells. Data are representative of three independent experiments, and the values represent the means ± standard deviation. *, *p* < 0.05; **, *p* < 0.01; ***, *p* < 0.001 versus the vehicle-treated control.

First, we confirmed that PFD treatment suppresses the growth of fibroblasts. PFD treatment (0.3 mg/mL) for 72 h suppressed the growth of commercially available prostate stromal cells (data not shown). Using these experimental conditions, we treated the PCa cells (LNCaP, E9, F10, AIDL, and PC-3) with PFD and found that PFD treatment suppressed the growth of all cell lines (Figure 1A). Among the LNCaP cells and sublines, growth suppression was more pronounced in LNCaP and E9 cells than in F10 and AIDL cells. We also assessed TGFβ1 secretion from PCa cells because of its relationship to cell cycle and apoptosis. TGFβ1 levels were measured in the culture medium of the PCa cells using ELISA. TGFβ1 secretion was significantly increased by PFD treatment in all PCa cells evaluated (Figure 1B). Among the LNCaP cells and sublines, the increase in TGFβ1 secretion was greater in LNCaP and E9 cells than in F10 and AIDL cells.

### 3.2. Pirfenidone Antiproliferative Mechanisms in LNCaP and PC-3 Cells

**Figure 2:**
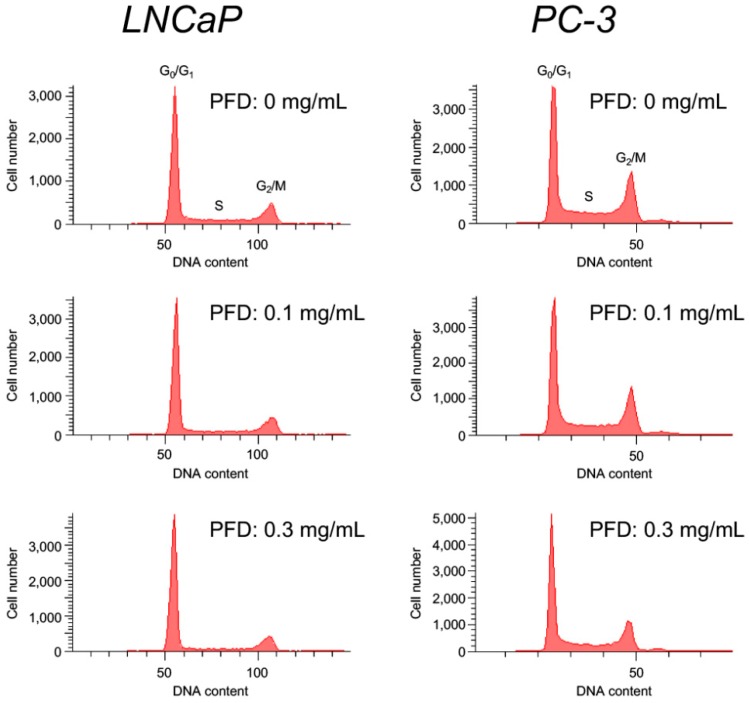
Cell cycle analysis by flow cytometry of human prostate cancer cells treated with pirfenidone (PFD). The cell cycle was determined by propidium iodide (PI) staining, as detailed in the “Material and Methods” section. The proportions of cells in the G_0_/G_1_, S, and G_2_/M phase were calculated from one representative experiment (*n* = 3).

**Figure 3:**
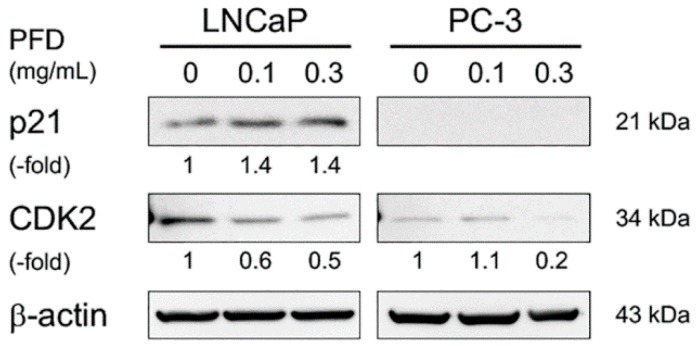
Effects of pirfenidone (PFD) treatment on the expression of cell cycle-related proteins in human prostate cancer cells. Both LNCaP and PC-3 cells were plated in 100 mm dishes and treated with PFD for two days. Cell lysates (50 µg) were separated by electrophoresis using a 12.5% SDS–polyacrylamide gel. After separation, the proteins in the gel were transferred to a polyvinylidene difluoride membrane by electroblotting. p21 and CDK2 protein levels were determined by Western blot analysis using specific antibodies. Equal loading of the samples was confirmed by measuring β-actin protein levels.

To investigate whether PFD treatment affects the cell cycle, we performed flow cytometric and Western blot analyses of cell-cycle regulatory proteins. In both LNCaP and PC-3 cells, PFD treatment increased the population of cells in the G_0_/G_1_ phase, which was accompanied by a decrease in S/G_2_ phase cells (Figure 2, Table 1 and Table 2). p21 protein expression was increased by PFD treatment in LNCaP cells, but was not detected in PC-3 cells (Figure 3). Of note, PFD-increased p21 protein expression was the highest in E9 cells (Figure S1). In contrast, CDK2 protein expression was clearly decreased in both PFD-treated LNCaP and PC-3 cells. Of note, PFD treatment did not induce early apoptosis in either LNCaP or PC-3 cells.

### 3.3. Effects of Pirfenidone Treatment on Androgen Receptor Signaling-Related Protein Levels in LNCaP and PC-3 Cells

**Figure 4:**
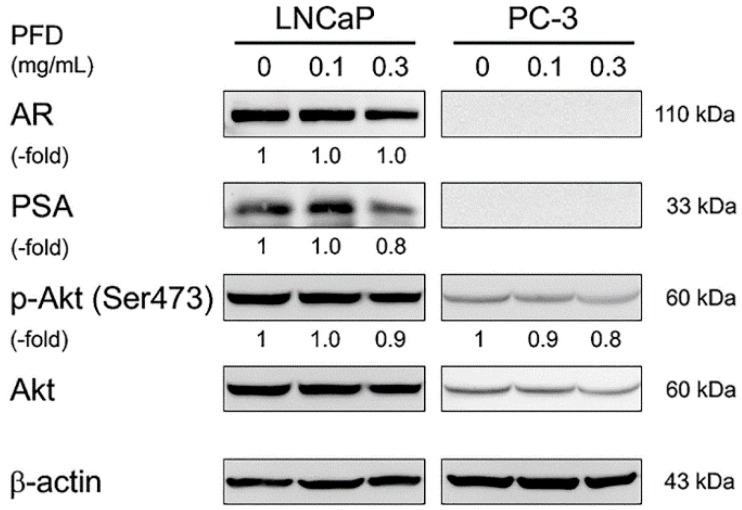
Effects of pirfenidone (PFD) treatment on androgen receptor signaling-related protein levels in human prostate cancer cells. Both LNCaP and PC-3 cells were plated in 100 mm dishes and treated with PFD for an additional two days. Cell lysates (50 µg) were separated by electrophoresis using a 12.5% SDS–polyacrylamide gel. After separation, proteins in the gel were transferred to a polyvinylidene difluoride membrane by electroblotting. Androgen receptor, prostate-specific antigen, phospho-Akt (Ser473), and total Akt protein levels were determined by Western blot analysis using specific antibodies. Equal loading of the samples was confirmed by measuring β-actin levels.

To confirm specific inhibition of PFD treatment on the AR signaling pathway, we evaluated the protein levels of AR and PSA in LNCaP cells by Western blot analysis. AR protein expression was not changed, but PSA protein expression was decreased by PFD treatment (Figure 4). In both LNCaP and PC-3 cells, PFD treatment slightly decreased the level of phospho-Akt (Ser473), suggesting slight inhibition of Akt phosphorylation. Of note, AR and PSA protein expression was not detected in PC-3 cells as reported previously [20].

**Figure 5:**
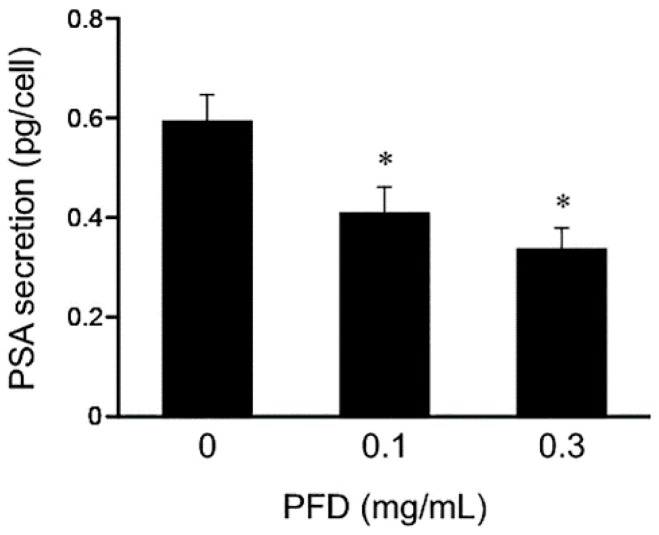
Effects of pirfenidone (PFD) treatment on prostate-specific antigen (PSA) secretion from human prostate cancer cells. The level of PSA secreted from LNCaP cells was determined by measuring the PSA level in LNCaP conditioned medium by ELISA. Values represent the means ± standard deviation. *, *p* < 0.05 versus the vehicle-treated control.

We further evaluated the effects of PFD treatment on PSA secretion by measuring PSA protein levels in conditioned medium from PFD-treated LNCaP cell cultures using ELISA. The PSA protein level was significantly reduced in LNCaP cell culture medium, suggesting that PSA secretion was inhibited by PFD treatment (Figure 5).

## 4. Discussion

Drug repositioning of the anti-fibrotic compound PFD to cancer treatment is not a novel idea, but investigation of the effects of PFD on PCa progression, considering the extracellular-matrix-rich microenvironment of PCa, would provide meaningful information for this potential application of PFD. In this study, we demonstrated that PFD treatment suppressed the growth and induced G_1_ cell cycle arrest in various PCa cell lines that differed in androgen sensitivity, suggesting that PFD may target not only fibroblasts but also heterogeneous PCa cells within the tumor microenvironment.

In animal models of fibrosis, PFD induced anti-fibrotic effects mainly via inhibition of TGFβ signaling in fibroblasts. TGFβ is a multifunctional cytokine that regulates cell proliferation, extracellular matrix production and degradation, cell differentiation, and apoptosis [29]. In animal models of fibrosis, PFD treatment inhibited fibrosis, which was associated with down-regulation of TGFβ, platelet-derived growth factor, and collagen synthesis in various types of cells, including human lung fibroblasts [30], rat hepatic stellate cells [14], human pancreatic stellate cells [31], rat renal fibroblasts [32], human Tenon fibroblasts [15], and rat cardiac fibroblasts [33]. 

In contrast, our results showed that PFD treatment significantly increased TGFβ secretion from all PCa cells evaluated, regardless of androgen sensitivity. Our previous study reported that TGFβ1 secretion from PCa cells was quite low compared with that from fibroblasts, especially carcinoma-associated fibroblasts [27]. TGFβ participates in cell proliferation and differentiation not only in normal processes such as embryonic development and wound healing, but also abnormal processes such as cancer progression and angiogenesis [34]. Although a number of studies have investigated the role of TGFβ, the results are still controversial. Importantly, the TGFβ signaling pathway is involved in both tumor-suppressive and tumor-promoting roles. The presence of TGFβ in the tumor microenvironment may promote tumor growth by enhancing stromal support and angiogenesis and by impairing immune surveillance [35]. In contrast, TGFβ plays a tumor-suppressive role by inducing G_1_ cell cycle arrest in various cell types, such as epithelial, endothelial, and hematopoietic cells and fibroblasts [36]. Cell-cycle inhibition by TGFβ is mediated in part by the up-regulation of antiproliferative proteins such as p15^INK4b^, p21^CIP1^, and p27^KIP1^. In this study, the p21^CIP1^ protein level was increased in PFD-treated LNCaP cells. Increased expression of p21^CIP1^, a cell-cycle-inhibitory protein, is not only associated with cell cycle inhibition, but also cell differentiation and senescence [37]. 

Lin et al. reported that the Akt pathway is associated with AR activation in LNCaP cells [38]. Iguchi et al. demonstrated that inhibition of Akt phosphorylation by the PI3K inhibitor LY294002 reduced PSA expression in LNCaP cells [39]. Previous studies reported that PFD treatment inhibits phosphorylation of Akt in rat hepatocytes [40], human lung fibroblasts [30], and human Tenon fibroblasts [16]. Similarly, in this study, PFD treatment slightly inhibited the phosphorylation of Akt in both LNCaP and PC-3 cells. In addition, PFD treatment reduced PSA protein expression and secretion in LNCaP cells. 

Serum PSA levels are influenced by a number of drugs, such as non-steroidal anti-inflammatory drugs and statins [41,42]; for example, the serum PSA level was found to be lower in aspirin users than non-users [41]. In contrast, Iguchi et al. reported that betamethasone, an agonist of the glucocorticoid receptor, increased PSA mRNA expression in LNCaP cells [43]. Similar to PFD, the antidiabetic drug troglitazone, which is a ligand of peroxisome proliferator-activated receptor gamma, reduced PSA expression in LNCaP cells [7]. Previous studies and our results suggest that PFD treatment reduces PSA expression, which is associated with inhibition of Akt phosphorylation in LNCaP cells. 

The tumor microenvironment of the prostate is highly complex and heterogeneous, and is composed of carcinoma-associated fibroblasts as well as epithelial cancer cells that infiltrate into the surrounding tumor stroma, referred to as reactive stroma [44]. This heterogenous stromal component of the prostate contains multiple populations of fibroblasts that are associated with tumorigenesis [45,46]. In this study, we demonstrated that the anti-fibrotic drug PFD suppressed the growth of human PCa cells by inducing G_1_ cell cycle arrest. In addition, our data suggest that PFD-induced growth suppression occurs independently of androgen sensitivity. Therefore, PFD may provide a novel therapeutic option for targeting not only fibroblasts surrounding cancer cells, but also heterogeneous PCa cells of varying androgen sensitivities within patients with CRPC.

## 5. Conclusions

In our studies of drug repositioning, we demonstrate that PFD may serve as a novel therapeutic drug that induces G_1_ cell cycle arrest in human PCa cells independently of androgen sensitivity. 

## Figures and Tables

**Table 1:** Effects of pirfenidone (PFD) treatment on cell cycle progression in LNCaP cells.

PFD (mg/mL)	Phase (%)		
	G_0_/G_1_	S	G_2_/M
0	61.8 ± 0.9	19.2 ± 0.3	17.8 ± 0.5
0.1	64.9 ± 0.7 **	16.8 ± 0.7 *	17.2 ± 0.5
0.3	72.3 ± 0.7 ***	12.0 ± 0.2 ***	14.7 ± 0.5 **

*, *p* < 0.05; **, *p* < 0.01; ***, *p* < 0.001 versus vehicle-treated control.

**Table 2:** Effects of pirfenidone (PFD) treatment on cell cycle progression in PC-3 cells.

PFD (mg/mL)	Phase (%)		
	G_0_/G_1_	S	G_2_/M
0	45.5 ± 1.3	20.5 ± 0.4	26.7 ± 0.7
0.1	51.1 ± 0.7 **	16.1 ± 0.1 **	25.5 ± 0.3
0.3	55.6 ± 0.6 **	15.2 ± 0.9 **	23.0 ± 0.9 **

**, *p* < 0.01 versus vehicle-treated control.

## Supplementary Materials

The following are available online at http://www.mdpi.com/2077-0383/8/1/44/s1, Figure S1: Effects of pirfenidone (PFD) treatment on the expression of cell cycle-related proteins in human prostate cancer cells. E9, F10, and AIDL cells were plated in 100-mm dishes and treated with PFD for 2 days. Cell lysates (50 µg) were separated by electrophoresis using a 12.5% SDS–polyacrylamide gel. After separation, the proteins in the gel were transferred to a polyvinylidene difluoride membrane by electroblotting. p21 and CDK2 protein levels were determined by Western blot analysis using specific antibodies. Equal loading of the samples was confirmed by measuring β-actin protein levels.
