# Supplementary material for: Pirfenidone, an Anti-Fibrotic Drug, Suppresses the Growth of Human Prostate Cancer Cells by Inducing G1 Cell Cycle Arrest
Source: J Clin Med. 2019 Jan 4;8(1):44. doi: 10.3390/jcm8010044 (PMC6351920; doi:10.3390/jcm8010044)
Supplement: Supplementary file 1 [file jcm-08-00044-s001.pdf]

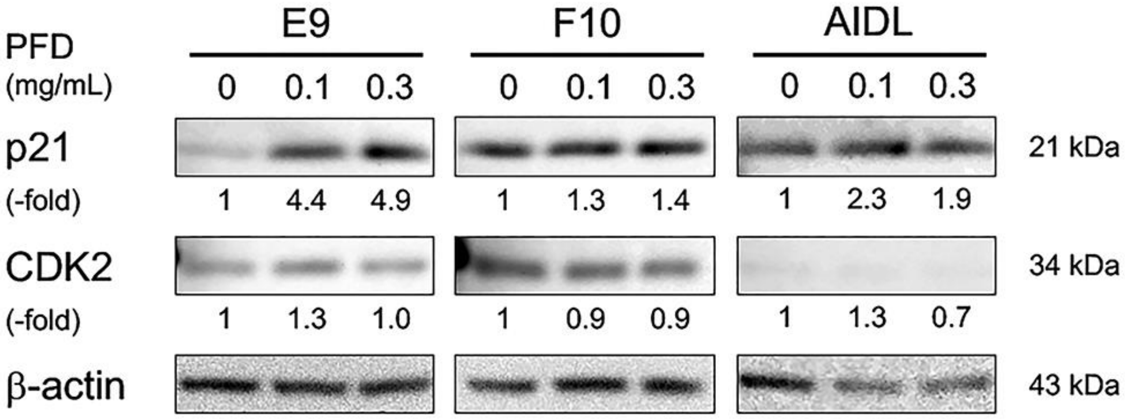

Figure S1. Effects of pirfenidone (PFD) treatment on the expression of cell cycle-related proteins in human prostate cancer cells. E9, F10, and AIDL cells were plated in 100-mm dishes and treated with PFD for 2 days. Cell lysates (50 μg) were separated by electrophoresis using a 12.5% SDS–polyacrylamide gel. After separation, the proteins in the gel were transferred to a polyvinylidene difluoride membrane by electroblotting. p21 and CDK2 protein levels were determined by Western blot analysis using specific antibodies. Equal loading of the samples was confirmed by measuring β-actin protein levels.
